# Recent Biomedical Approaches for Chitosan Based Materials as Drug Delivery Nanocarriers

**DOI:** 10.3390/pharmaceutics13040587

**Published:** 2021-04-20

**Authors:** Andreea Teodora Iacob, Florentina Geanina Lupascu, Maria Apotrosoaei, Ioana Mirela Vasincu, Roxana Georgiana Tauser, Dan Lupascu, Simona Eliza Giusca, Irina-Draga Caruntu, Lenuta Profire

**Affiliations:** 1 Department of Pharmaceutical Chemistry, Faculty of Pharmacy, Grigore T. Popa University of Medicine and Pharmacy of Iasi, 16 University Street, 700115 Iasi, Romania; andreea.panzariu@umfiasi.ro (A.T.I.); florentina-geanina.lupascu@umfiasi.ro (F.G.L.); apotrosoaei.maria@umfiasi.ro (M.A.); ioana-mirela.vasincu@umfiasi.ro (I.M.V.); roxana.tauser@umfiasi.ro (R.G.T.); dan.lupascu@umfiasi.ro (D.L.); 2 Department of Morphofunctional Sciences, Faculty of Medicine, Grigore T. Popa University of Medicine and Pharmacy of Iasi, 16 University Street, 700115 Iasi, Romania; simona-eliza.giusca@umfiasi.ro

**Keywords:** chitosan, nanoparticles, nanofibers, nanogels, liposomes, nanocarriers, drug delivery systems

## Abstract

In recent decades, drug delivery systems (DDSs) based on nanotechnology have been attracting substantial interest in the pharmaceutical field, especially those developed based on natural polymers such as chitosan, cellulose, starch, collagen, gelatin, alginate and elastin. Nanomaterials based on chitosan (CS) or chitosan derivatives are broadly investigated as promising nanocarriers due to their biodegradability, good biocompatibility, non-toxicity, low immunogenicity, great versatility and beneficial biological effects. CS, either alone or as composites, are suitable substrates in the fabrication of different types of products like hydrogels, membranes, beads, porous foams, nanoparticles, in-situ gel, microparticles, sponges and nanofibers/scaffolds. Currently, the CS based nanocarriers are intensely studied as controlled and targeted drug release systems for different drugs (anti-inflammatory, antibiotic, anticancer etc.) as well as for proteins/peptides, growth factors, vaccines, small DNA (DNAs) and short interfering RNA (siRNA). This review targets the latest biomedical approaches for CS based nanocarriers such as nanoparticles (NPs) nanofibers (NFs), nanogels (NGs) and chitosan coated liposomes (LPs) and their potential applications for medical and pharmaceutical fields. The advantages and challenges of reviewed CS based nanocarriers for different routes of administration (oral, transmucosal, pulmonary and transdermal) with reference to classical formulations are also emphasized.

## 1. Introduction

The development of polymeric drug delivery systems (DDSs) using the latest nano-technology approaches had gained scientists’ attention in the pharmaceutical field, the most used polymers being those of natural origin such as chitosan, starch, cellulose, gelatin, elastin and alginate [1]. The end of the 1970s coincides with the first research targeting polymeric drug DDSs, when the development of polymethacrylate-based NPs was carried out in order to increase the immunogenicity of antigens [2]. The high interest in natural polymers is supported by several advantages of this type of polymers such as biodegradability, biocompatibility, inexpensiveness, easy availability and biological effects [3]. The polymeric drug carriers used in DDSs have an important role in preventing the degradation of drugs, improving the drugs’ pharmacokinetic and pharmacodynamics profile, increasing the degree of absorption and penetration at specific target tissues [4].

*Chitosan as a promising natural polymer for the formulation of DDSs.* Chitosan (CS) is an ideal candidate for DDSs because it is non-toxic, biocompatible, biodegradable, low imunogenic and bioadhesive. Furthermore, its structure is similar to collagen and can be used to mimic the extracellular matrix [5]. In addition, CS proved to have bacteriostatic, fungistatic, haemostatic hypobilirubinaemic, hypocholesterolemic [6], antiacid and antiulcer, anti-inflammatory, antioxidant, antidiabetic and neuro-protective effects [7]. Based on these special characteristics, CS is a promising biomaterial and has attracted increasing attention of researchers for developed CS-based materials for biomedical applications, especially as DDSs [5]. The CS based DDSs are used for the delivery of proteins/peptides, growth factors, anti-inflammatory drugs, antibiotics, anticancer drugs, vaccines and so forth; as well as in gene therapy [8,9]. CS is a linear hydrophilic polyelectrolyte polysaccharide composed of β-(1–4)-linked d-glucosamine and *N*-acetyl-d-glucosamine, obtained by alkaline or enzymatic *N*-deacetylation of chitin, the second most abundant polymer in nature, after cellulose [10]. Chitin is present in the exoskeleton of crustaceans (crab, lobster, squid, shrimp), insects and fungal strains [11]. The hydroxyl and amino groups present in the structure of CS are responsible for its chemical reactivity, solubility and bioactivity [3]. CS has low solubility at physiological pH but in dilute acidic solutions the primary amino groups are protonated and CS becomes positively charged and water-soluble [5]. The positive charged also explains the interaction of CS with negative charged microorganism cell membranes and so its antimicrobial effects [12].

An important feature of CS is its mucoadhesive ability, which is explained by electrostatic interactions between the positively charged amino groups of polymer chain with the negatively charged mucin glycoproteins residues, rich in sialic and sulfonic acids [13]. CS based nanocarriers have many advantages due to their smaller size and increased surface area, mucoadhesive properties and tight junction modulation capacity. They can be used for targeting drugs to cells and tissue through the capacity to penetrate into cells by endocytosis or receptor-mediated transcytosis, which increases the delivery of the molecules into the cells, can enhance the drug stability, achieve controlled releasing drugs or decrease the cytotoxicity of drugs [8,14].

CS, either alone or as composites, are suitable substrates in the fabrication of different types of products like hydrogels, membranes, beads, porous foams, nanoparticles, in-situ gel, microparticles [15]. The formation of the self-assembled CS-based nanostructures which display outstanding features, such as high protein encapsulation ability and prolonged drug release profile, is also reported [16].

**Figure 1:**
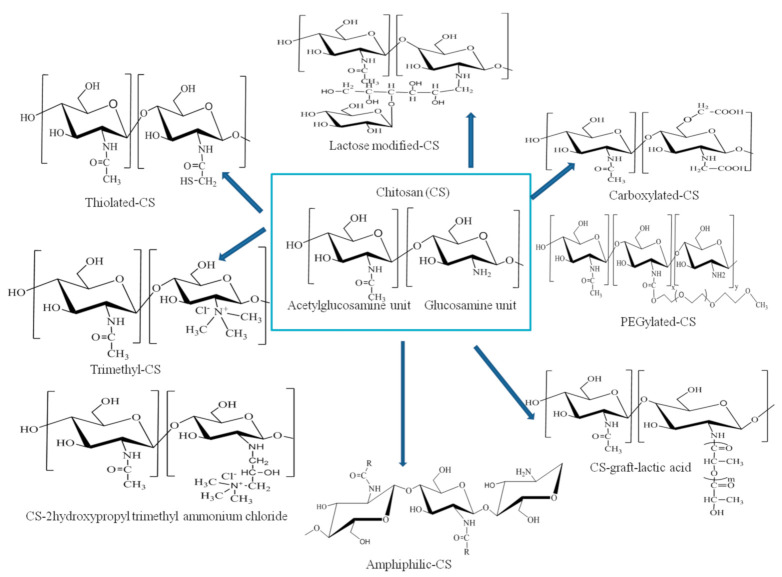
CS derivatives resulted by chemical modification of CS.

*Chemical modifications of CS for the development of suitable CS-based DDSs.* In order to improve the characteristics of CS, different derivatives were synthesized, based on the chemical reactivity of hydroxyl (primary or secondary) and primary amine groups [17]. Chemical modification of CS results in the formation of quaternized-CS, thiolated-CS, carboxylate-CS, amphiphilic-CS, CS with chelating agents, PEGylated-CS and lactose modified-CS [18] (Figure 1). For example, by trimethylation of the primary amino group of CS, the cationic character increases substantially and its mucoadhesive properties consequently increase [15]. In the case of thiolated-CS, disulfide bonds with mucus glycoproteins of the mucus gel layer and intra-chain disulfide bonds are also formed, which increase the mucoadhesive properties [15]. Using gamma irradiation from a ^60^Co source, safe and biocompatible membranes based CS and HEMA (2-hydroxyethyl methacrylate) were also formulated [19,20].

**Figure 2:**
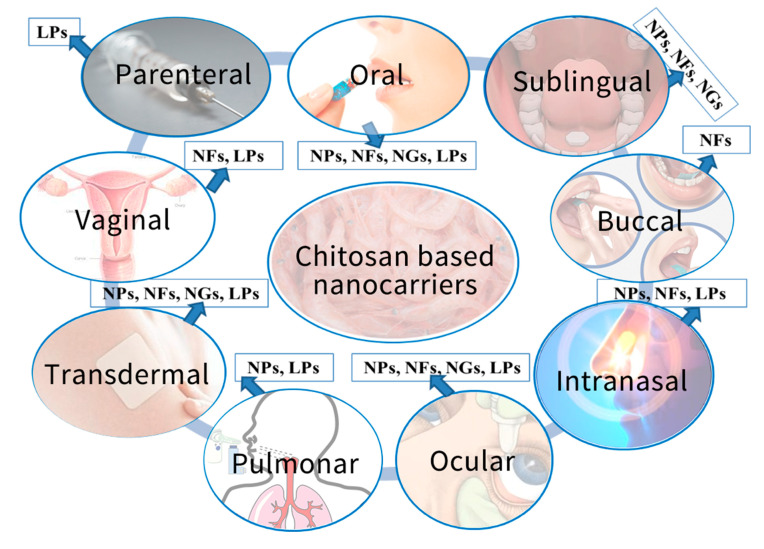
CS based nanocarriers for biomedical applications.

The aim of this review is to emphasize the versatility of CS in the formulation of different DDSs, based on the recently developed CS nanocarriers such as nanoparticles (NPs), nanofibers/nanoscaffolds (NFs), nanogels (NGs) and liposomes (LPs), targeting especially oral, transmucosal, pulmonary and transdermal administration (Figure 2). The advantages and challenges of the reviewed CS based nanocarriers with reference to classical formulations are also highlighted.

## 2. Types of Chitosan Based Nanomaterials

**Figure 3:**
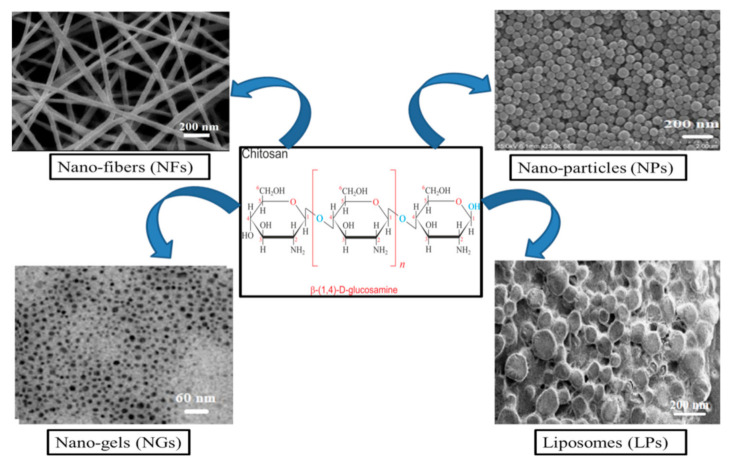
Representation of the main CS based nano-materials with biomedical applications.

The nanomaterials, especially NPs, NFs, NGs and LPs (Figure 3), due to their nano-size dimension, unique surface and operational/functional features, can surmount manyof the hindrances from conventional DDSs and are therefore ideal candidates that can be used in different biomedical applications. 

### 2.1. Chitosan Based Nanoparticles (CS-NPs)

Nanoparticles (NPs) are attractive DDSs, based on their ability to load therapeutic small drugs, peptides, ribonucleic acids and so forth, being appropriate candidates for controlled drug release at different target sites such as corneal, nasal, transdermal, intravenous and gastro-intestinal mucosa. NPs delivery systems have many advantages such as long-circulation and controlled release of drugs, improved drug solubility and stability, enhanced efficacy and reduced toxicity [21].

Due to their smaller size (average size below 1000 nm) and increased surface area, NPs are characterized by increased selectivity and specificity to a defined target and have the capacity to cross the cell barrier by endocytosis even receptor-mediated transcytosis, which leads to improved release of molecules inside the cells [22]. NPs can also undergo surface modification that can make it feasible to add specific molecules that target certain cells, for example tumor tissue [23].

The preparation methods of the NPs include ionic gelation, emulsion, reverse micellar method, coacervation, precipitation, nanoprecipitation and the sieving method [7,18]. Chitosan based nanoparticles (CS-NPs) act as excellent carriers due to their properties such as hydrophilicity, non-toxicity, biodegradability, biocompatibility and bioadhesivity.

The formation of CS-NPs is influenced by several parameters such as the concentration, the molecular weight (Mw) and the degree of deacetylation (DD) of CS, the crosslinking agent concentration, zeta potential (ZP) of NPs and so forth. It was reported that by increasing the Mw of CS there is an increase in the viscosity of the polymeric solution, obtaining particles with a larger size [24,25]. Therefore, CS with low and medium molecular weight (LMw, MMw) and low concentrations are recommended for obtaining NPs with a small size. The drug release (DR%) profile from the CS-NPs matrix is also improved by reducing the size of NPs, due to the positive values of ZP and high loading efficiency (DLE%) [26]. Generally, the DLE increases with the increase of drug concentration until the maximum charged concentration is reached [27]. The DD of CS influences its solubility in aqueous acid media and so the oral bioavailability; the increase of DD being associated with a good absorption capacity [28]. 

The physical stability of the NPs in solution is measured by ZP, the extreme positive and negative values being correlated with high repulsive forces, which contribute to colloidal stability [25,29,30]. 

The most important characteristics of CS-NPs, in terms of particle size, drug loading efficiency (DLE%), drug release (DR%) profile from CS-NPs matrix and zeta potential (ZP) values as well as of CS features, are presented in Table 1.

### 2.2. Chitosan Based Electrospun Nanofibers (CS-NFs)

The electrospun nanofiber scaffolds properties mimic the nanoscale characteristics of the native extracellular matrix (ECM) and provide a specific response, useful for the treatment of different disorders. The advantages of nanofibers are numerous, and in addition to the controlled release of drugs, these systems can improve the solubility and permeability of embedded drugs due to their porous structure and satisfactory surface/volume ratio [33,34].

The most common and widely used method to produce nanofibers (NFs) is the electrospinning method. This technique is very useful for preparing sub-micron or nano-scale fibers because it offers several advantages; it is a facile and a cost-effective method, it is easy to embed bioactive principles into the nanofibers, and it does not require heating during the obtaining process, which is of key importance principally for thermo-sensitive compounds [34]. The basic mechanism involved consists of the high electrostatic forces which surmount the surface tension of a polymer droplet, existing at the nozzle tip. These charged drops are formed from the spinneret results in modeling in the shape of Taylor’s cone, the ejection of a straight fluid jet which, in the end, leads to nano-sized fibers formation, collected on the oppositely charged collector [35]. For the formation of functional polymers nanofibers, soluble/insoluble drug particles and nanoparticles of drugs/liposomes can be added to electrospinning solution [36].

Regarding the polymeric solution, its viscosity and conductivity affect the diameter and the diameter distribution of NFs in a noticeable way, being crucial factors in the stretching of the charged jet [37]. These parameters are closely related to the nature and concentration of the polymer. If the concentration of the polymer solution is low, the applied electric field and surface tension can make the tangled polymeric chains disaggregate into shreds before reaching the collector. The stretching in the whipping region due to the surface charges draws the fluid jet into the nano-scale [38]. The solvent used in the electrospinning process must also completely dissolve the polymer and have a moderate boiling point for obtaining suitable NFs [39].

The electrospinning of CS is a difficult process due to the very high viscosity of the solution in low concentration, caused by its polyelectrolytic nature [40]. Many studies showed that the addition of nonionic polymers (poly-ethylene oxide, poly-vinyl-alcohol) to the CS solution leads to better electrospinnability, by improving the viscoelastic characteristics, giving rise to uniform NFs [41,42].

Recent data revealed that many therapeutics such as anticancer, antibiotics and bio-actives (genes, cells, enzymes, growth factors) were successfully loaded into CS based NFs [43].

The most important characteristics of CS–NFs, in terms of drug content (DC) and drug release (DR%) profile from CS-NFs matrix, as well as of CS features, are presented in Table 2.

### 2.3. Chitosan Based Nanogels (CS-NGs)

Nanogels (NGs) are hydrogels in nanoscale, with three-dimensional polymer networks, formed through physical or chemical crosslinking, with an ability to retain a large volume of water and with the advantage of not dissolving in an aqueous environment [49]. The CS’s properties, such as biocompatibility and biodegradability combined with the properties of NGs, such as flexibility and deformability, size, large surface area, soft nature, controllable stability, high loading capacities and easy functionalization make CS-NGs promising active agents for drug delivery and also for bioimaging, cell culture and therapy [50,51]. Several methods, such as ionotropic gelation, physical gelation, polymerization, self-assembly and microemulsion, have been reported for the preparation of CS-NGs [51].

CS has the ability to gel itself to form NGs, without any surfactant, solvent or cross-linker, due the presence of the –OH and –NH_2_ groups leading to the capability of forming electrostatic interactions and hydrogen bonds between the polymer chains [51,52]. These CS-NGs contain only water and CS with size ranging from 0.2 nm to 1000 nm. Some characteristics of CS such as Mw, DD degree, viscosity and concentration of polymeric solution can significantly contribute to particle size distribution and also influence the thermo sensitive features of NGs. It was reported that the NG structure is depending on formulation type, either covalent or ionic crosslinking [53]. The –OH and –NH_2_ groups can also form covalent bonds in the presence of a chemical linker [51]. In addition, the protonation of –NH_2_ groups at low pH contributes to the positive charge of CS and to electrostatic interactions with the components of the mucus or with the epithelial surfaces that are negatively charged. These types of interactions provide mucoadhesion properties and sustain the use of CS-NGs as mucoadhesive drug delivery systems. 

The CS-NGs can act as carriers, protecting the drugs from degradation and/or elimination. The hydrophilic nature of the CS-NGs limits their use to hydrophilic drugs delivery. The entrapment of lipophilic drugs into the polymeric matrix of CS, in the form of NGs, can take place by the binding of hydrophobic groups, such as alkyl, cholesterol, acyl, 5β-cholanic acid, cholic acid and deoxycholic acid, to the CS molecule. Furthermore, hydrophobic CS derivatives, such as carboxylate CS and CS sulfate, has an important role in conferring an amphiphilic or amphoteric character, which leads to interactions with both anionic and cationic molecules and also give the ability of self-assembling and water-solubility [54,55].

The most important properties of the NGs are related to the fact they can be designed as responsive NGs drug delivery [56]. In the presence of the regular environmental factors, including temperature variations, ionic strength, light, pH, reducing reactions, intracellular enzymes such as cytochrome P450 (CYP450), the NGs act smart, changing in their physiochemical properties such as volume, water content, refractive index, network permeability and hydrophilicity [57]. These stimuli can be natural, in the body, or can be applied from outside the body in order to guide a nanocarrier to the specific target or to be activated it at a specific tissue [57]. The applicability of the stimuli-responsive NGs includes mainly cancer or inflammation conditions [58]. The chemical functionalization of CS with a targeting ligand (e.g., galactose, folic acid for tumor cell target) was also proposed for targeted drug delivery, especially anticancer drugs delivery [59]. The mucoadhesive CS-NGs were designed, mainly for periodontal delivery systems (flurbiprofen CS-NGs, triclosan CS-NGs, doxycyline CS-NGs) buccal delivery (farnesol CS-NGs), ocular delivery (acetazolamide CS-NGs) or transdermal delivery (terbutaline CS-NGs) [60,61]. In addition, these CS-NGs may undergo modifications using pH-sensitive groups or specific ligands in order to release therapeutic agents such as anticancer drugs, genes and vaccines at specific targets [62].

The most important characteristics of CS-NGs, in terms of drug content (DC)/drug loading efficiency (DLE%) and drug release (DR%) profile from CS-NGs matrix, as well as of CS features, are presented in Table 3.

### 2.4. Chitosan Coated Liposomes (CS-LPs)

Liposomes (LPs) are spherical vesicles formed by an inner aqueous phase protected by one or several concentric phospholipid bilayers. These nanocarriers present some specific features such as: embedding hydrophobic or hydrophilic drugs, ameliorating some properties of the incorporated drugs (for example, solubility or toxicity) or protecting the degradation of the substances. LPs can also improve the penetration through tissue, the capacity of site-specific targeting and possess self-assembly property [62,68,69].

Among the advantages of LPs are biocompatibility, the ability to reduce the side effects of some drugs and improving the pharmacokinetics and drug target sites’ bioavailability. Moreover, LPs are safe because they are similar tothe biological membranes [62,68].

Related to the particle size and vesicle lamellarity the LPs are unilamellar, multilamellar and multivesicular membranes. The last type is designed for the parenteral route, while the first two ones can be used for various routes, including oral. The quantity of drug embedded is affected by the vesicle bilayers size and thus the half-life circulation can be influenced [68].

The methods by which LPs can be obtained are thin-lipid film hydration, freeze–thawing, reverse-phase evaporation, dehydration–rehydration, solvent injection, double emulsion, fast-extrusion, and detergent-depletion methods. Microfluidic and supercritical reverse phase evaporation techniques have been also used [70].

The composition of LPs can modify the binding ability, the distribution, and the drug release. The most important characteristics of the liposome surface are fluidity, charge, and permeability. The liposomal surface can be changed by conjugation to polymers and/or ligands in order to design more efficient DDSs. The polymer coating can modulate the stability of LPs and so increase the bioavailability, the pharmacokinetic and pharmacodynamics profiles [13].

CS based liposomes (CS-LPs) are often obtained by coating, which allows the change of the liposomal surface properties. It is considered that the CS coating may improve the structural rigidity, the integrity of membranes and the stability through electrostatic interactions. The increasing concentrations of CS can improve the rigidity and stability of LPs because allow the absorption of polymer chains through liposomal surface. When the concentration gets to a saturation point, this effect is maximum and after this value begins to appear some aggregations of CS chains and some not regulated tangles. Thus, it can destabilize the liposomal structure and the membrane fluidity raise [71].

An important parameter for obtaining suitable CS-LPs is the CS/lipid ratio, by increasing this ratio, stable LPs, slightly larger in size and with enhanced storage stability and positive ZP, can be obtained [72]. The DR (%) profile from the LPs matrix is a complex one that depends on several factors, such as the physico-chemical properties of the drug (hidro or liposolubility), the liposomal membrane integrity, the diffusion of the drug through phospholipid layers or fluid bilayers, the thickness of CS layers and so forth [73]. It was noted that the free, positively charged amino groups of CS are responsible for of positive ZP values of CS-LPs, and the ZP value increases with the thickness of the CS layer, which leads to increased LPs stability by increasing electrostatic forces between particles [74,75,76]. The percentage of encapsulation for the hydrophilic drugs is conditioned by the volume of the aqueous phase inside the liposome particle. Consequently, by increasing the concentration of lipids there is an improvement in the percentage of incorporation of hydrophilic drugs [72].

The main disadvantage of CS coating is related with its reduced solubility at physiological pH. The formulations-based CS could also more easily release the embedded drugs, having an important swelling degree in aqueous media [9]. For this reason, the most used are CS derivatives such as carboxylated, quaternized, thiolated and amphiphilic. The most important carboxylated derivatives are carboxymethyl and N-succinyl CS. Carboxymethyl CS presents improved solubility and biocompatibility, has proved pH-sensitivity and nontoxicity, and is often used for various types of drug delivery systems [77]. Quaternized derivatives of CS are used to improve the solubility, to decrease the aggregation and to increase the stability of LPs. A known representative is trimethyl chitosan, which has been used to cover LPs for oral and ocular formulations [13]. Chitosan-thioglicolic acid is included in thiolated CS derivatives, which has proved to be an efficient nanocarrier. The most important advantage of this system is related to the mucoadhesive properties that are assigned to thiol groups. This polymer system can also enhance the permeation of embedded drugs and thus the oral bioavailability [78]. The amphiphilic CS derivatives could be obtained by N-acylation or amidation reactions. One example could be the synthesis of *N*-octyl-*N*-arginine-CS, by grafting hydrophilic arginine groups and hydrophobic octyl groups to the amino group of CS in order to increase the bioavailability [79]. Recent studies revealed that is more effective the grafting of hydrophilic scaffolds as polyethylene glycol or sulphate groups [80].

These studies evidenced that CS-LPs can be used as nanocarriers in various areas of drug delivery such as nasal, pulmonary, oral, ocular, transdermal and parenteral systems. These delivery systems can incorporate different drugs including peptides, proteins, hormones, anticancer and antimicrobial drugs, vaccines, genes, enzymes, and siRNA [13]. 

The most important characteristics of CS-LPs, in terms of LPs size, drug loading efficiency (DLE%), drug release (DR%) profile from CS-LPs and zeta potential (ZP), as well as CS features, are presented in Table 4.

## 3. Chitosan Based Nanomaterials as Oral Nanocarriers

Oral administration or per os (p.o.) route is one of the most preferable and convenient means of drug delivery, intended to have a systemic effect. Generally, the traditional oral drug delivery systems have proven to have limited bioavailability caused by several factors, depending of the drugs’ features, such as the fast gastric emptying due to low solubility of the drug in the elevated pH environment, the intense enzymatic degradation of drug at gastro-intestinal tract (GIT) or its degradation in the colon, the absorption of the drug in the upper side of the GIT or short half-life, and respectively narrow absorption window for some drugs [82,83]. In order to overcome these limitations, scientists have focused their attention on the new formulation such as gastro-retentive drug delivery systems (GRDDS), in which CS plays a major role [83].

The release mechanism of drugs from the CS matrix consists of swelling of the polymer matrix, drug diffusion through the pores of polymeric matrix, polymer erosion and degradation. In addition, the drugs release from these formulations is dependent on the pH value, so in gastric media (pH 1.6) the release will be more then in intestinal medium (pH 6.5). It is possible to appear an initial burst release, explained through swelling capacity of the polymer and creating pores in polymer matrix, or diffusion of the drug that may exist on the polymer surface [11,18]. The CS matrix can suffer surface modification at –NH_2_ and –OH active groups of CS, changes that lead to improvement the solubility in intestinal media, the muco-adhesiveness, the drug encapsulation efficiency and the stability of CS [18]. 

### 3.1. CS-NPs as Oral Nanocarriers

By development of NPs, the disadvantages of oral drug administration can be overcome, because the NPs prevent the drugs enzymatic degradation and increase the GIT stability of acid-instable drugs. In addition, NPs can adhere to the GIT due to mucoadhesive properties, leading to release the drug for a longer time [22]. 

To enhance the oral bioavailability of paclitaxel, a new NPs formulation based on CS cysteine and polylysine have been developed. Each component has a certain role; therefore, cysteine can increase the mucoadhesive ability and permeation effect of CS; polylysine, with positive charge, can increase the intestinal permeation of paclitaxel by strengthening the electrostatic interaction with negatively charged integrin receptors to extending the residence time. In vivo therapeutic efficacy of this formulation was performed on a Heps tumor cells mice model [84].

Doxorubicin, an anticancer drug with a broad spectrum, has proven effective in treating some animal and human tumors. Unfortunately, this drug at a high dose causes cardiotoxicity and myelosuppression. Adimoolam et al. [85] synthesized in situ CS stabilized magnetite NPs with doxorubicin which have proven effective as drug carrier and target specific release, all this leading to suppression of cancer cells without affecting the healthy cells. The mechanism of releasing doxorubicine in target tumor cells consists ofcreating the sensitive imine bond to the endosomes and lysosomes or to increase the pH specific to the tumor cells. The release of doxorubicin from this polymeric matrix was 90% at pH 4.6, which ensures the possibility of drug delivery to the intracellular components of the cancer cells [85].

Insulin remains the mainstay of treatment for diabetes and obtaining insulin formulations for oral administration is a highly desired goal. Subcutaneous administration is considered awfully invasive and associated with a lot of risks and the oral bioavailability of insulin is very low due to its high molecular weight, enzymatic degradation and the molecule instability in gastric acid environment. However, the oral delivery of insulin is the preferred route for diabetics because it is easy to administer, imitates physiological insulin release, improves glucose homeostasis and avoids the displeasure of insulin regular injection. Insulin-loaded NPs were obtained via self-gelation with CS and snail mucin. This formulation proved a good NP encapsulation efficiency and a release profile over a period of 8 h [86]. Another approach was to obtain a formulation in which insulin is incorporated into thiolate CS-NPs. This formulation has great muco-adhesive properties due to disulphide bond formation involving cysteine-rich domains of mucus glycoproteins. This mucoadhesion increases their residence time in the gut, prolongs insulin release and enhances its bioavailability into the blood [87]. 

Olanzapine, a thieno-benzodiazepine derivate, is an antipsychotic from the second-generation with fewer side effects, particularly extrapyramidal symptoms. Recently, olanzapine-loaded NPs have been developed as a possibility to improve the pharmacological and pharmaco-toxicological profile of olanzapine. CS-coated- olanzapine-loaded NPs engineered for oral route by Veragten et al. [88] showed great mucoadhesion properties, superior drug encapsulation with a good drug release rate. This formulation has also shown a good ability to restore prepulse inhibition (PPI) disruption induced by apomorphine on rats [88].

Several studies have been performed to enhance the oral absorption of enoxaparin, an anticoagulant used in various disorders. This drug has a very low oral absorption rate due to its high molecular size, negative charge, high water solubility, low absorption through intestinal wall and first pass effect. The development of solid lipid NPs loaded with enoxaparin-saturated fatty acid conjugates has proven to be a good strategy to improve the oral bioavailability of enoxaparin. Dong et al. [89] developed new polymer-lipid hybrid NPs with enoxaparin for oral delivery and the results of the experiment performed on rats, demonstrated that this formulation achieved the higher oral absorption of enoxaparin, which was 4.5 times higher compared to enoxaparin formulated as an oral solution [89].

### 3.2. CS-NFs as Oral Nanocarriers

Ranitidine hydrochloride is an H_2_ receptor antagonist highly susceptible to microbial degradation and which has variable absorption in GIT and a half-life of only 2–3 h. These limitations were minimized by the formulation of a floating GRDDS consisted in tripolyphosphate (TPP)-crosslinked NFs mats based on CS and polyethylene oxide [44]. The in vitro drug release studies, showed for the formulated NFs mats a decrease of the initial burst release and a sustained release profile for the ranitidine hydrochloride. Based on the results of the thermal analysis and tensile strength it was noticed that the crosslinking with TPP leaded to an increase in the thermal stability and mechanical characteristics and a decrease in swelling degree. The buoyancy test analysis showed also that the NFs mats remain floated onto surface of the dissolution medium more than 48 h, and the mucoadhesive strength analysis emphasize the remarkable bioadhesive strength [44].

AnjiReddy et al. [43] described the formulation of CS-NFs on donepezil as a faster drug release system used for Alzheimer’s treatment. After formulation, the NFs were evaluated in terms of in vitro dissolution, the antibacterial activity and the MTT cell viability assay. The donepezil loaded CS-NFs attained maximum drug release (80% drug release in the phosphate buffer solution, pH 6.8 within 360 s), compared with marketed oral disintegrated tablets with donepezil. The in vivo drug release study showed that the value of the mean plasma concentration of donepezil released from CSNFs administered to rats via oral route was almost 2 fold higher than the mean concentration of donepezil released from oral disintegrating marketed tablets and the drug release time was shorter. The NFs formulation provided a higher donepezil release compared with traditional dosage forms. The results demonstrated the rapid disintegration of NFs after oral administration, an advantage for increasing compliance in elderly patients [43].

A novel GRDDS systems based on CS-gellan NFs loaded with resveratrol was described as a potential gastro-intestinal cancer long-term prevention and treatment therapy. The encapsulation efficiency of resveratrol in these nanofibers was 86 ± 6% and the drug amount released in the gut was approximately 51%. Regarding the efficacy of this formulation in cancer therapy, studies have shown that resveratrol loaded CS-NFs have almost the same cytotoxicity against HT29 cancer cells compared to free resveratrol. The results regarding in vitro biocompatibility and cytotoxicity studies have shown that NFs formulation can be an excellent drug delivery carrier for resveratrol [90].

### 3.3. CS-NGs as Oral Nanocarriers

NGs are considered to be efficient oral drug delivery systems that have multiple advantages: they can increase the residence time of the drug in the GI tract, protect the active substance from degradation, ensure an efficient release from the polymer matrix and increase the absorption of drugs through GI membranes [63]. Unfortunately, NGs have some disadvantages that avoid their use in therapy: modest drug entrapment and low uptake, poor stability before achieving the target, biodegradability after achieving the target [57]. Positive charge of the CS is important for the transcellular transport of the drug, but the solubility of CS in the acid medium often limits its use for oral delivery, resulting in burst release of the drug at the stomach [91]. The insoluble CS derivatives are not very effective for improving the release of the embedded drugs. Furthermore, the use of the chemical cross-linker agents can affect the properties of the NGs in terms of low mechanical resistance, reduced control over hydrogel pore size and of toxicity of cross-linker [91,92]. 

Most studies on the oral delivery of drugs based CS-NGs refer to a local effect in gastrointestinal disorders, particularly inflammatory or tumor targets. Zhou et al. [93] prepared a novel anionic conjugate NG system, succinyl-glycol-CS-succinyl prednisolone, for targeting inflamed sites in ulcerative colitis. By functionalization of glycol CS-succinyl prednisolone conjugate NGs with succinic anhydride, the nanoparticles became negatively charged and preferentially were concentrated in target site, in the injured colonic mucosa, where proteins were found to be positively charged. In addition, the in vivo studies suggest that NGs released prednisone (PD) gradually and no toxic systemic effects of the PD were observed. The suppression of the colonic damage was better for NGs than for PD alone at 10 mg PD/kg [93].

Feng et al. [64] studied the transport mechanism of doxorubicine (DOX) embedded in CS/carboxymethyl-CS-NGs (CS/CMC-NGs) across intestinal epithelium. The experiments were performed in vitro on the Caco-2 cell model and in vivo on rats, to study the transport of CS/CMC-NGs throughout the intestinal barrier permeability. Excellent absorption of DOX-CS/CMC-NGs was observed throughout the entire small intestine by both paracellular and transcellular transport pathways. CS promoted the DOX absorption enhancement in the duodenum and CMC induced permeation enhancement in the ileum and jejunum. The mucoadhesive effects of CS/CMC-NGs were promoted in the paracellular transport of DOX and, possibly, NGs might be internalized by active uptake or transported over Caco-2 cells [64].

### 3.4. CS-LPs as Oral Nanocarriers

Cao et al. [94] reported the synthesis of CS-coated levodopa LPs and their effects on rat model induced dyskinesia. It was observed that the abnormal involuntary movements decreased significantly in the liposome group in comparison with the levodopa group and the dyskinesia that may appear in the advanced stages was also reduced [94,95]. 

For the development of a new system for insulin oral delivery, insulin-CS polyelectrolyte complexes associated with lecithin LPs was developed. It was revealed that the stability of insulin was increased by adding the insulin-CS polyelectrolyte complexes to lecithin liposomal dispersion. In vivo results, using a streptozotocin induced rat diabetic model, evidenced a decrease of blood glucose levels, related to subcutaneously administered insulin [95,96].

CS coated alendronate LPs were developed to improve the bioavailability and to decrease the gastric irritation of alendronate. These systems possessed high muco-adhesive characteristics and presented increased oral bioavailability of alendronate in rats. It was demonstrated that CS coating could increase the drug absorption and could also protect the degradation of LPs by digestive enzymes. Moreover, the liposome system could prevent the appearance of esophageal adverse effects that are related to alendronate oral administration. The results proved that the CS coated alendronate LPs is a more stable and more effective oral drug delivery system [97].

Zhou et al. [74] have presented the synthesis of CS-LPs of acteoside, for which an improvement pharmacokinetic profile, in terms of decreased in vivo release and increased stability and bioavailability, were evidenced. There were prepared carboxymethyl chitosan (CM-CS) and quaternary chitosan (TMC)-coated liposomes (CM-CS/TMC-LPs) embedded curcumin, in order to improve the oral bioavailability and the retention time in the systemic circulation of curcumin [77]. These systems proved to present favourable gastric acid tolerance and may be an encouraging nanocarrier with higher efficacy and safety than curcumin. It was observed that a larger concentration of curcumin was distributed in the liver, spleen and lungs.

New based CS-thioglycolic acid coated LPs embedded with paclitaxel and pluronic F127 were developed as stable and sustained-release drug delivery systems. The tests evidenced an increased potency of mucus adhesion, penetration through cell membranes, which lead to an extended residence time in gastro-intestinal tract. This combined drug delivery system proved to be more mucoadhesive and adherent to the intestinal mucosa in comparison with CS-thioglycolic acid coated LPs and could allow the intestinal epithelial cells passage into the systemic circulation in order to be used as nanocarrier for chemotherapy [98].

Novel CS covered ursolic acid (UA) LPs (CS-UA-PLs) were also reported for tumor cells targeting, good, controlled drug release and low side-effects. The studies performed in vitro (HeLa cells) and in vivo (mice bearing U14 cervical cancer) demonstrated that this formulation could be used in the localized tumor treatment and to decrease the total drug dose and side-effects. In addition, the CS-LPs presented mucoadhesive features and their oral bioavailability was improved through the prolonged release of the drug at the tumor site [99].

Some polyelectrolyte complexes formed by multilamellar vesicles (MLV) and polymer-inhibitor conjugate were synthesized. Protease inhibitor-aprotinin was covalently bound to CS. Biological evaluation included the determination of serum calcium after oral administration of calcitonin solution and two oral formulations: calcitonin embedded chitosan covered MLV or CS-aprotinin covered MLV. It was observed a more pronounced for calcitonin loaded CS-aprotinin MLV than CS coated MLV [100].

Gradauer et al. [101] evaluated the in vivo effects of thiomer-coated LPs (S-protected or unprotected CS-thioglycolic acid) encapsulated salmon calcitonin. These LPs have shown a good ability to penetrate the intestinal mucus layer, to have a favourable residence time and to increase the permeation effect on the mucosa. The blood calcium levels were intense reduced in case of LPs coated with S-protected thiomers, which support the increase of the bioavailability of orally administered calcitonin [101].

## 4. Chitosan Based Nanomaterials as Transmucosal Nanocarriers

Transmucosal administration offers multiple advantages, compared to the traditional enteral, intravenous or intramuscular routes. CS is an ideal candidate for transmucosal formulations because it has mucoadhesive properties and proved the ability to open the tight junctions between epithelial cells. Therefore, CS can promote the transport of macromolecular drugs and complex molecules such as proteins and peptides [102]. 

Administration through the buccal route consists of the application of drugs in the oral area in order to absorb and penetrate them into systemic circulation. The research on buccal DDSs had drawn attention because of its advantages: the rich vascularization of the buccal area, the increase of drug bioavailability through avoiding first-pass liver metabolism and the possibility of administering vulnerable substances at the GIT [103].

The *sublingual mucosa* represents a portion of the oral mucosa more penetrable than palatal and buccal mucosa due to the low degree of keratinization and of small mucosal thickness (100–200 µM). The very high concentration of blood and lymphatic vessels, along with its small thickness, makes it a favorable route for drugs for which rapid onset of action and immediate release are necessary [33]. The sublingual route has several advantages compared to the conventional route of administration. It also avoids the high acidic environment and enzymatic degradation that could damage the drugs administered and avoids the first pass metabolism. The *sublingual route* also has some disadvantages, which consist of a small surface area for drug absorption through passive diffusion and also the continuous washout of the drug due to salivary secretions, which limits the drug residence time in the oral cavity [104]. The common drug related barriers for sublingual administration are a low degree of lipophilia, a molecular weight higher than 500 Da., a dose administration higher than 10 mg and a pKa which does not allow the drugs to stay unionized at the pH salivary value (6.0). These disadvantages can be largely offset by mucoadhesive formulations that can improve the rate of drug absorption [104,105].

*The nasal route* is another mucosal route with topical drugs administration through which a systemic or a localized effect is achieved. Most of the nasal mucosal surface (~180 cm^2^) is highly vascularized and the microvilli existence at the apical region of cells increases the surface area for drug absorbance [106]. Among the advantages of the nasal route of administration we can enumerate: avoiding the first hepatic passage, rapid response to the action of drugs by rapid penetration into the systemic circulation, avoiding the discomfort created by parenteral administration and good patient compliance. The main disadvantages for intranasal administration are: removal of drugs through mucociliary clearance and nasal secretions, the presence of enzymes and peptidases in the mucus and the influence of drug properties such as molecular weight and lipophilia on penetration through the nasal mucosa. These challenges can be overcome through mucoadhesive systems [33,107]. Penetration through the nasal mucosa occurs through transcellular pathway, paracellular pathway or via trigeminal nerves. In the last years several nanocarriers for intranasal administration, targeting the brain, were designed and CS, based on its interesting properties, is very useful for this type of application [108].

*Ocular drug delivery* is also a favorite route of administration due to the ease of use and patient compliance. The ocular mucosa represents the layers that cover the conjunctiva and corneal surfaces and is constituted of a complex macromolecular structure of proteins, lipids, DNA and mucin [33]. Ocular delivery represents a challenge for the delivery of drugs due to some major anatomical and physiological barriers presented by the eye. The anatomical barriers consist of the blood-aqueous barrier, the strongly vascularized choroid, which allows the rapid clearance of drugs, the corneal barriers and the retinal pigment epithelium, which is also considered the blood-brain barrier equivalent of the eye. The physiological barriers include the presence of a mucin and tear layer which determine the rapid drug removal as a result of drainage of the fluid tears in the nasolacrimal duct induced by blinking [109]. 

The release rate, the required drug loading and the ocular retention time of drug delivery systems depend on the bioavailability, potency and clearance of the drug at the desired site. The limitations of the loading capacity with active substances are due to the properties of the materials used and the limiting size of the absorption surface at the ocular level [110].

In the last few years, CS formulations for ocular delivery, especially NPs and LPs, have been extensively researched. CS exerts numerous beneficial properties for ocular administration, such as: high degree of biodegradability with extensive mucoadhesive characteristics and minor immune response as a result of its capacity to link Toll-like receptors. Another useful property of CS for administration to the ocular mucosa is that it can form viscous solutions through water dispersion, preventing drainage after administration. Of the CS derivatives, CS-iminothiolane represents an important category of mucoadhesive polymers that form disulphide bonds between the free thiol groups of the derived polymer and the cysteine residues present in the mucin [33]. For improving the mucoadhesive properties, CS in association with other carriers (poly(lactic-*co*-glycolic acid or alginate), as a dual system, was also used. In vivo tests support the claim that CS contributes to corneal wound repair by enhancing keratinocyte pro healing functions, which leads to rapid onset in collagen synthesis [18,111]. 

*Vaginal mucosa* could also be used as a transmucosal route. It is characterized by four different highly vascularized layers with ease of accessibility and which avoid the first pass effect of the drug and enable self-administration [107,112]. Drawbacks of using the vaginal route include: age-related hormonal activity, condition of the mucosa thickness and production of vaginal fluid and enzymes; the presence of cervico-vaginal fluid, which can lead to drug removal; some probability of degrading some drugs unstable to the acidic pH of the vaginal fluid and not ultimately the physicochemical characteristics of the drugs; solubility, pKa, and molecular weight, affecting the drug solubility in the vaginal fluid, chemical stability and tissue penetration [33].

**Figure 4:**
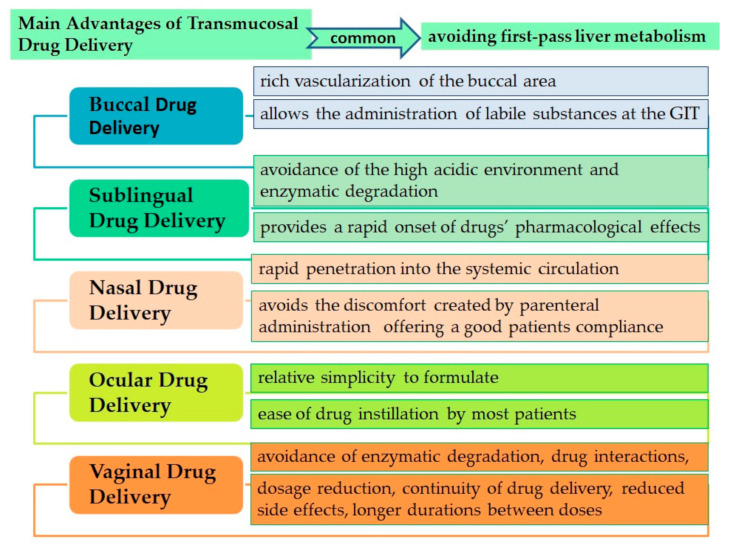
Representation of the main advantages of the transmucosal DDSs.

The main advantages offered by the transmucosal route compared to the traditional ones (oral, intravenous or intramuscular) are illustrated in Figure 4. 

### 4.1. CS-NPs as Transmucosal Nanocarriers

Insulin was prepared as NPs with thiolated *N*-triethyl-CS for buccal delivery. This formulation protects insulin from harsh conditions in the GIT, increases its residence time on the absorption area and enhances drug penetration through the oral mucosa. Among the advantages of this formulation are a good mucoadhesion and penetration improving the effect of CS through the tiolation process. This effect occurs by creating disulfide bonds between the thiol groups of the chitosan derivative and cysteine sequences present in the mucus glycoproteins. Enhanced mucoadhesiveness can be a good method for surmounting the disadvantages of administration on the buccal mucosa such as mucus turnover, poor available area of oral mucosa for drug absorption, and the drug dilution process by the presence of saliva. An in vivo experiment developed on rabbit buccal mucosa showed excellent insulin permeation through buccal mucosa, the insulin permeability reaching 96% in 480 min [113]. To increase the bioavailability of insulin, insulin-loaded CS-NPs for nasal administration have been also engineered. The in vivo data showed that insulin CS-NPs produced a decrease of basal blood glucose levels with 52.9% or 59.7% in the rat and with 72.6% in the sheep, much better than that obtained after the use of nasal insulin CS solution (40.1% in the rat, 53.0% in the sheep) [114]. 

A mucoadhesive CS-NPs entrapping the chemotherapeutic oxaliplatin was engineered by Matos et al. for tumors’ oral mucosa [115]. The approach of this study was the increasing of the penetration effect using the iontophoresis process as a way to obtain a rapid drug permeation through the oral mucosa to transport hydrophilic molecules in biological tissues. This formulation increased by3 fold the capacity of drug penetration compared with a simple drug solution and, moreover, by applying the iontophoresis process the amount of oxaliplatin transported into the porcine mucosa increased 2 fold. CS-NPs loaded with oxaliplatin were effective in killing cancer cells of an oral tumor cell line [115].

The nose-to-brain delivery is a new strategy to transport drug loaded NPs through the biological brain barrier. Using this strategy, pramipexole was encapsulated into CS-NPs, to deliver the drug via nose to brain for the treatment of neurological disorders, using the induced rat model of Parkinson’s disease. The cumulative rate of drug penetration through the nasal mucosa for pramipexol CS-NPs was 83.03% ± 3.48 after 24 h. In vivo anti-Parkinson’s activity revealed the brain targeting potential of pramipexol CSNPs, as compared to the results obtained for the solution-type formulation of that drug [116].

For enhancing the bioavailability of desvenlafaxine, an antidepressant drug, CS-NPs was developed for nose-to-brain delivery. Nowadays, antidepressants are administered mainly orally, although their therapeutic efficacy is closely related to the ability to cross the blood-brain barrier to reach the central nervous system. This new nose-to-brain drug delivery system consists of polylactide-*co*-glycolide (PLGA) and CS-NPs loaded with desvenlafaxine. The results showed that this formulation enhanced levels of neurotransmitters serotonin and noradrenaline considerably compared with the depressed control. Further, it was found high levels of desvenlafaxine both in systemic circulation and in brain. Therefore, the results of this study support the claim that nose-to-brain delivery is an effective way to treat depression and other brain disorders [117]. 

Bhattamisra et al. [118] prepared rotigotine loaded CS-NPs to enhance the drug concentration in the brain via intranasal delivery. Rotigotine is a dopamine agonist, used for the treatment of Parkinson’s disease with highly efficient but low bioavailability. The nasal administration of rotigotine loaded CS-NPs allowed brain targeting to be increased and so the bioavailability of the drug, compared to only rotigotine. The results of the pharmacokinetic study support the claim that the intranasal route is the best route for direct transport of rotigotine to the brain [118]. 

Carboxymethyl-CS was used for obtaining carbamazepine loaded NPs to improve its uptake to the brain via nose to brain delivery. Carboxymethyl-CS is a water-soluble derivative, which is easy to obtain and has an amphoteric character with many possible applications. Carbamazepine has the autoinduction function and an increase of its levels in the liver leads to an increase of CYP3A4 activity leading to a significant increase in drug clearance, conducting to a low half-life. This drug has a narrow therapeutic range (4–12 g/mL), so, fluctuations in the concentration of the drug in the blood could lead to many side effects. Following the experiments performed, it was observed that a sufficient amount of drug reached the brain through nose to brain drug delivery, leading to a higher efficacy, with decreasing dose and side effects [119].

In a study conducted by Chhonker et al. [120], CS-NPs loaded with amphotericin for treating fungal keratitis were engineered. Amphotericin B is an antifungal antibiotic available in the present in a lyophilized form for intravenous use and as eye drops. The drawback of this drug is its hydrophobic character. For increasing the solubility in water, sodium deoxylcholate, a water soluble surfactant was added to an ophthalmic solution. Unfortunately, the instillation is painful owing to surfactant and increased frequency of administration leading to low patient compliance. In addition, a low drug residence at the precorneal surface happened, which leads to decreased bioavailability. CS-NPs loaded with amphotericin B seem to overcome these disadvantages. The in vivo data showed that NPs were much more efficient than the commercial formulation; the bioavailability increased ∼2.04 times more and precorneal residence time ∼3.36 times more. Therefore, this formulation has the following advantages: an increased retention time of the drug on the precorneal mucosa and a prolonged drug release [120].

In another study, CS-NPs coated with hyaluronic acid (HA) for dexamethasone release into the eyes was developed. These CS-NPs offer, on the one hand, a sustained topical dexamethasone delivery into the eyes and, on the other hand, protect the drug from effective enzymatic degradation. HA was used to make NPs discrete, free-flowing and to improve their mucoadhesive characteristics [121]. 

Another NPs formulation based on CS and HA has been developed to increase the bioavailability of erythropoietin, a neuroprotective and neuroregenerative drug, due to its biological properties, such as suppressing cellular apoptosis and reducing inflammation, oxidation and excitotoxicity [40,122]. Erythropoietin also has an important role in preventing the retinal ganglion cells apoptosis; today, being available some formulations for administration on the following routes: systemic, intravitreal and retrobulbar routes. Recent studies investigated erythropoietin delivery to the retina through subconjunctival administration. For improving erythropoietin ocular bioavailability after topical administration, NPs were developed by ionotropic gelation using a different CS-HA ratio. The in vivo study conducted on fresh porcine corneas, scleras and conjunctivas and revealed that CS-HA (1:1) NPs enhanced erythropoietin residence time and penetration through the different ocular layers [122].

Yu et al. [123] manufacturing a cerium oxide loaded glycol CS-NPs for scavenging reactive oxygen species (ROS), which have been incriminated as major factors in dry eye development. In vitro and in vivo studies have shown that this formulation alleviated ocular surface disease with improvement of dry eye disease by stabilizing the tear film and maintaining the epithelium integrity. The NPs improved the solubility of cerium oxide, so a loading up to 80% was achieved. The cerium oxide loaded glycol CS-NPs showed a considerable decrease of intracellular ROS in cornea and conjunctiva with improving tear volume in dry eye mice models. There was also an improvement in symptoms and a cessation of pathological changes at the cellular and molecular levels [123].

### 4.2. CS-NFs as Transmucosal Nanocarriers

NFs, due to its flexibility and its unique surface properties such as viscosity, mucoadhesive properties and ease of application, offer a good substitute for other mucoadhesive films or patches via buccal route administration for both local and systemic drug delivery [107]. 

Fast dissolving oral films (FDOFs) based on CS/pullulan NFs were assessed as a buccal drug delivery system. Encapsulating aspirin in the FDOFs for oral mucosal release was performed in order to avoid several side effects of aspirin to the gastric mucosa: ulceration, gastric mucosal erosion, or even gastric perforation. The CS-NFs based FDOFs can achieve uniform distribution of the poorly water-soluble drug-aspirin and the result is a faster dissolution of aspirin due to hydrophilic characteristics of CS. Thermal stability and glass transition temperature of FDOFs were enhanced with the addition of CS and water solubility test showed that the FDOFs can be completely dissolved in water within 60 s [103]. 

Chen et al. [124] describes the formulation based on self-formed liposome and core/shell NFs using hydrosoluble bio-adhesive polymers, such as carboxymethyl-CS and sodium carboxymethyl cellulose embedded with carvedilol, as a buccal mucosal delivery system, using poly(vinyl alcohol) (PVA) and poly(vinyl pirrolydone) (PVP), respectively, as film forming agents. The combination of self-assembled liposome from electrospun NFs and soluble bonding polymers was performed in order to increase the carvedilol’s buccal absorption. After scanning electron microscopy and confocal laser scanning microscopy investigation it was determined the core/shell arrangement as follows: carboxymethyl cellulose/PVA and carboxymethyl-CS/PVA were concentrated in the shell layers and the core stratum was represented by a mixture of PVP, phospholipids and carvedilol. The fibers dissolution in the first 2 h had a linear silhouette without drug bursting release, and the carvedilol was discharged in a joined Fickian diffusion–erosion way. The permeability tests throughout porcine TR146 cell culture and buccal mucosa revealed that the self-constituted liposome structure together with the bio-adhesive polymer enhanced the drug penetration. Indirect cytotoxicity assay revealed that TR146 cells were relative safe after incubation with the extraction medium of NFs at concentration below of 10 mg/mL [124].

Another substance taken into consideration for a better release and permeation across buccal mucosa ex vivo was insulin, embedded in a structure of chitosan/PEO NFs in HFP (1,1,1,3,3,3-hexafluoro-2-propanol) [125]. For the in vitro determination of insulin release rates, using an ELISA experiment proved that elevated chitosan: PEO ratio at electrospinning process leads to faster insulin release and smaller fibers. Insulin permeation across the buccal mucosa using porcine transbuccal model, demonstrated that CS:PEO 20 blend ratio nanofibers have 16 times higher buccal permeability compared with free insulin. These findings imply that CS-based NFs have the potency to act as an efficient transbuccal insulin delivery carrier [126].

An interesting study was performed on migraine treatment with the two most commonly used drugs, sumatriptan succinate and naproxen salts, incorporated into NFs. In vitro assays performed on these drugs revealed very high dissimilarities regarding the sublingual permeability rates, meanwhile the rates of both substances were augmented several times using CS-NFs, as the drug carrier when compared to drug solutions [45]. Because the drugs permeate preferentially as non-ionized moieties, CS-NFs incorporating both the drugs in non-ionized forms have been developed. These NFs proved to be mechanically resistant, very flexible, and with a drug load ability of up to 40% of membrane mass, advantages that could be used for sublingual drug delivery systems development [45].

Mašek et al. [127] describes the formulation of a CS-PEO nanofibrous reservoir layer in order to elaborate proper product for mucosal application in a non-invasive way, especially for sublingual and buccal tissues [127]. This type of nanocarrier consists of three important parts: an electrospun nanofibrous reservoir layer, a mucoadhesive film coating and a protecting backing stratum (PEGATEX S 30 g/m^2^), each of the layers with different roles. The mucoadhesive film layer will assure a good grip of the entire system to the oral/sublingual mucosa after application, while the nanofibrous reservoir stratum will function as a stockpile for dendrimers, polymeric and lipid-based NPs, virosomes, virus-like particles or LPs. The nanofibrous reservoir stratum due to its tremendously large surface area allows increased degrees of NP loading, which they can be furthermore either conversely assimilated to the surface of NFs or they can be placed in the pores between the NFs mats. In order to demonstrate this concept, a porcine model was used for the lymph-node delivery of PLGA-PEG NPs trans-/intra-mucosal. The in vivo and ex vivo pig model demonstrated the application of NFs mucoadhesive films as protecting nanoparticle reservoirs used for a controlled and sustained delivery of nanoparticles into draining lymphatic node and submucosal tissue areas. The future applications of these systems could be in the formulation of non-invasive sublingual vaccines and as well in the development of “printed vaccine technology”, such as influenza and papilloma virus [127].

Jahantigh et al. [47] have presented the preparation of CS-NFs containing nano-encapsulated *Shigella* subunit antigen, N-IpaD (N-IpaD/NFs) as novel intranasal vaccine delivery biodegradable system. The antigen-containing CS nanofibrous mat was formulated by chitosan/AcOH solution electrospinning and it showed an acceptable loading capacity for proteins. The potential of this novel carrier for the nasal delivery of *Shigella* subunit vaccine was examined on guinea pigs. The guinea pigs intranasal immunized by administration of N-IpaD/NFs indicated elevated mucosal and serum antibody reaction when compared with those of other groups. It was also found that when subjected to wild-type *S. flexneri* 2a in a keratoconjunctivitis Sereny test the N-IpaD/NFs group was protected. Intranasal administration of encapsulated *Shigella* subunit antigen in CS-NFs considerably increased the systemic and local immune responses, compared to intramuscular or intranasal administration of soluble *Shigella* subunit vaccine, which means that the CS-NFs are a promising vehicle for intranasal delivery of *Shigella* antigens [47].

A transparent and nano-structurally stable biomimetic chitosan/collagen-hyaluronate based nanofibrous membranes as wound dressings for corneal chemical injury treatment, have been also developed [128]. The SEM analysis showed the arrangement of chitosan in the outer layer, demonstrating the role of a coating surface agent, while as the nanofibrous core consists of a collagen-hyaluronate. These membrane NFs showed high mechanical and biological properties compared to human amniotic membrane and were convenient to the selective adhesion of corneal, conjunctival cells and fibroblasts. The in vivo evaluation in rats on an alkali-burned corneal damage model indicated an improved re-epithelialization in corneal tissue within one week for the group treated with the biomimetic electrospun NFs [128].

CS based electrospun NFs mats also represent useful ocular inserts for the delivery of ophthalmic drugs, as shown by Mirzaeei et al. [103], who develop an ocular delivery system for delivery of triamcinolone acetonide. Due to the small diameter and homogeneity, the prolonged and controlled release profile of triamcinolone acetonide, this CS formulation, can suppress the disadvantages of the regularly used ocular delivery systems [129].

The drugs delivery via the vaginal mucosa utilizing drug-incorporated electrospun NFs is an innovative strategy and beneficial in cases that needs a sustained local drug delivery [33]. Traditional vaginal formulations cannot preserve efficient drug concentration for extended periods of time and in consequence, novel vaginal formulations based on NPs or electrospun NFs, have obtained growing attention [130]. For local chemotherapy of cervical cancers in mice, the fabrication of cisplatin-loaded poly (ε-caprolactone)/chitosan NFs as a vaginal mucoadhesive drug-delivery system was reported in a small scale study by Aggarwal et al. [33]. This study proved a sustained release profile for up to 1 month, and also an improved antitumor activity in vivo. The predominant factors establishing the drug sustained release pattern of the prepared formulation are: mucoadhesive property of NFs, limited degradation of poly-caprolactone under vaginal physiological conditions and low water solubility. In vivo activity was validated using an orthotopic cervical cancer model in Swiss albino mice established by inducing at cervix region of the mice in the vaginal mucosa, the Erlich ascites carcinoma cell lines. Intracervical administration of the cisplatin loaded NFs showed smaller % cell viability compared against the plain drug and an enhanced anti-tumor efficiency in animals at 14th and 21st day after the initiation of treatment [46].

Electrospun CS based NFs co-loaded with doxorubicin and indocyanine green, as anticancer agents, were developed by Wang et al. [131]. The formulation was carried out in two steps: first the preparation of doxorubicin and indocyanine green co-loaded mesoporous silica nanoparticles (DIMSNPs) was performed, followed by the nanoparticles’ incorporation into CS/PVA scaffold to shape multifunctional composite NFs via the electrospinning process. Using conditions that imitate the vaginal environment, the nanofibrous mats reported a site-specific drug release as compared with the local delivery of a thermo-sensitive DIMSNPs-loaded gel. In vivo evaluation was studied on mice in both subcutaneous and orthotopic cervical cancer models, where it was concluded that the drug permeation in the hard nodular tumor was very challenging. Another good finding of this study refers to the tumor inhibition rate for orthotopic cervical/vaginal cancer which presented a value of 72.5%, demonstrating its huge ability to be analyzed further as a viable alternative in the treatment of cervical cancer [131].

### 4.3. CS-NGs as Transmucosal Nanocarriers

Based on the mucoadhesion of CS, Aminu et al. [60] have developed new NGs formulation with triclosan (antibacterial drug) and flurbiprofen (anti-inflammatory drug) for local treatment of periodontitis. The triclosan poly-ε-caprolactone NPs were embedded onto CS-NGs loaded flurbiprofen. This formulation was designed to enhance the triclosan solubility and to improve the residence time of flurbiprofen. It was suggested that the molecules of drugs are physically adsorbed onto the surface of the NGs at the same time as entrapped within its inner structure, leading to wider particle size of 150–400 nm. The formulation showed pH-dependent swelling and erosion, temperature-responsiveness and it was characterized by a strong bioadhesivity. The in vivo study using a rat model of periodontitis proved high antibacterial and anti-inflammatory effects for NGs when compared with drugs physical mixtures [60].

Nikoomanesh et al. [132] designed mucoadhesive CS-NGs for delivery of farnesol, an antifungal drug. By encapsulation of farnesol onto CS-NGs (88% farnesol loaded), the farnesol’s inhibitory action against *C. albicans* together with the down-regulated Rim101 and SAP6 genes expression was improved [132].

A recent study combined the advantage of CS-NGs, in terms of simplicity of application and the extended remanence time with the significant permeation power of surfactant (Span 60) based nanovesicles, in designing of a new formulation of acetazolamide-nanovesicles loaded onto CS-NGs, for ocular delivery. The CS-NGs exhibited good muco-adhesion time, less irritant action and an increased and significantly prolonged release profile for acetazolamide (only 38.3% after 6 h) compared with acetazolamide-nanovesicles formulation, where the drug was released rapidly (64.9% after 6 h) [133].

### 4.4. CS-LPs as Transmucosal Nanocarriers

New intranasal LPs loaded with fexofenadine to treat allergic rhinitis have been developed [134]. It was reported that CS improve the stability of fexofenadine and the LPs system allowed a higher adsorption to mucin, which leads to the ability of retaining for a prolonged time in the nasal cavity. The increasing of the bioavailability of fexofenadine in comparison with oral administration has also been reported. Ghrelin containing LPs formulation embedded with CS were obtained for administration via nasal route for cachexia treatment. Ghrelin is a hormone with peptide structure and an appetite stimulation effect which is rapidly degraded. Using the liposome formulation, the mucoadhesion of ghrelin was increased, the residence in the nasal cavity was also prolonged, and hence its uptake to the brain was improved [135]. Pralidoxime chloride loaded cationic CS-LPs, have been prepared for intranasal administration, targeting the brain damages. This formulation showed a significant reduction of brain damage and death of rats after poisoning by paraoxon-induced acetyl-cholinesterase inhibition [136]. The development of some chitosomes (S region of hepatitis B antigen encapsulated liposomes) as nasal vaccine delivery vehicle for eliciting viral specific humoral, mucosal and cellular immune responses have been also reported [137].

Various retinal diseases (including macular edema) could be conducted to permanent retinal structural damage and threatens vision. High biocompatibility, non-immu -nogenicity, corneal penetration and prolonged clearance times are the main advantages of LPs as drug carriers for ocular application. A new eye drop formulation based on CS-LPs, able to release triamcinolone acetate to the retina as non-invasive and safe drug administration system, has been developed [138]. A good penetration through the corneal mucosal barrier and an accumulation in vitreous body when their efficacy was assessed using a choroidal neovascularization model were observed [139]. Li et al. [140] reported the synthesis of CS coated LPs loading cyclosporine A or diclofenac sodium. Both systems proved to present prolonged drug retention, enhanced drug permeation and biocompatibility [140,141]. A similar system was developed for timolol maleate, for which a significant mucin adhesion and retention at the corneal surface for a longer period of time, compared with commercial eye drops, were noted. An improved corneal permeation and good ocular tolerability were also reported [142]. CS is known for its antimicrobial and antifungal effects. It is considered that the mucoadhesive properties of chitosan could prolong the retention at the vaginal site and act against biofilms responsible for high recurrence of infections. The results of in vitro studies referring to CS-LPs loading clotrimazole evidenced a prolonged release of clotrimazole by comparison with control. The permeation of CS-LPs through the vaginal tissue was decreased when compared with non-coated liposomes and assured increased retention. In addition, the non-irritability and safety of these systems were proved by conducting in vivo experiments realised on pregnant sheep [143]. In the same manner were developed chitosomes loading metronidazole for the localized therapy of mixed vaginal infections and have evidenced that can be active on *C. albicans* [144].

## 5. Chitosan Based Nanomaterials as Pulmonary Nanocarriers

Pulmonary route has gained outstanding interest as a potential approach to obtain both local and systemic effects. The advantages of the pulmonary route include: rapid onset of action, high efficacy alongside with the lack of first pass metabolism. The fast onset of therapeutic action is due to the thin absorption barrier, high tissue vascularity and large surface area of the lungs. For inhalation drugs to reach the systemic circulation they must overcome certain impediments: the alveolar lining fluid, proteolytic degradation, lung surfactant, epithelial cells and macrophage clearance. In the last years the inclusion of drugs in nanocarriers for inhalation administration becomes an encouraging research direction in order to increase the local bioavailability and aerosol performances [145].

### 5.1. CS-NPs as Pulmonary Nanocarriers

CS-NPs have been vastly investigated as a carrier for lung drug delivery, due to mucoadhesive properties, a high adherence to the lung mucosa, their capacity to open the intercellular tight junctions of the lung epithelium with the improving of drug uptake. CS-NPs loaded with rifampicin, an antitubercular drug, were formulated by ionic gelation for direct targeting to the lungs, in order to increase efficacy and to scale down the side effects of rifampicin [146]. In vitro drug release assays of formulated NPs indicated sustained drug delivery up to 24 h. The pharmacokinetic study of rifampicin NPs showed an enhanced maximal plasma concentration, prolonged residence time and slow the clearance of rifampicin from the lungs compared to market formulation and conventional dry powder inhalation [146].

For lung cancer therapy, siRNA is also an attractive strategy that can be administered both intravenously and through inhalation. The bioavailability of siRNA when administered by the lung is low because of the intracellular barrier and the development of new polymer carriers of siRNA for pulmonary administration is a real challenge. Ni et al. [147] developed siRNA NPs using baclofen functionalized trimethyl-CS, as polymeric carriers to augment the uptake of siRNA via the liaison with GABAb receptor. siRNA was successfully loaded into NPs with proper aerodynamic features for deep lung accumulation and a prevention of siRNA serum-induced degradation (a integrity of siRNA maintained about 77.34% under the protection of the polymer). The in vitro cell apoptosis and gene silencing tests showed that the gene expression of unfunctionalized NPs was reduced up to 62.89% [147].

Heparin, a linear anionic polysaccharide, has an anticoagulant effect, which exhibits poor bioavailability when orally administered. Currently, it is administered via the parenteral route, but the pulmonary route has drawn notable attention as a potential approach to delivering useful quantities of the anticoagulant. Heparin-loaded CS-NPs were formulated for systemic delivery upon pulmonary administration. Both the size and encapsulation efficiency of heparin loaded CS-NPs depended on the acidic or neutral media in which they were prepared. Thus, those prepared in acidic media had a size of 156 nm and encapsulation efficacy of 100% and those prepared in neutral environment had the size of 385 nm and encapsulation efficacy of 43%. The results of in vivo assay emphasized that the formulation of heparin loaded CS-NPs was efficient at delivering heparin in the bloodstream after pulmonary administration and those prepared in neutral conditions resulted in a notable prolongation of the coagulation time in comparison to the control [148].

Bedaquiline is a novel anti-tuberculous (anti-TB) drug, a diaryl-quinoline compound that acts via a new mechanism by inhibiting the enzyme necessary for energy generation in bacteria (proton pump of ATP synthase) [149]. In order to reduce the treatment period and to reduce the dosage of administration and the adverse effects, CS-NPs have been developed to target specific delivery and prolonged release of this drug into the lungs. The results of cell viability assays indicated insignificant toxicity with about 90% cell viability for CS-NPs loaded with bedaquiline. In vivo pharmacokinetics study showed superior concentration of bedaquiline in lungs via the developed formulation (C_max_ 4302.52 ± 234.12 μg/g), compared with conventional dry powder inhaler formulation (C_max_ 3384.13 ± 490.30 μg/g)) and oral drug solution (C_max_ 2602.57 ± 308.91 μg/g). The chronic toxicity assay in various organs also corroborated a better safety profile of CS-NPs loaded with bedaquiline [149].

### 5.2. CS-LPs as Pulmonary Nanocarriers

Rifampicin loaded chitosan-coated liposomes (CS-LPs) were developed by Zaru et al. [150] for drug-delivery to the lungs by nebulisation. It was noted that liposomes cytotoxicity was reduced and the stability of the formulation during nebulisation was high. The mucoadhesive characteristics, which are required for a better drug-delivery to the lungs, were also improved [150]. Manca et al. [151] have developed dry powder for inhalation based on chitosan/carrageenan-coated rifampicin loaded liposomes. The polymer coating layer-by-layer with carrageenan and CS could increase the stability of the liposomal surfaces and also the adhesion on airways and epithelial cells. Rifampicin loaded uncoated and coated vesicles proved to reduce basal alveolar epithelial cells derived from adenocarcinoma with pulmonary localization. It was considered that coated liposomes presented a high capacity to be easily internalized and so evidenced a prolonged residence time when compared with uncoated liposomes [151].

CS-coated LPs loading *N*-acetylcysteine were also prepared in order to obtain a formulation with prolonged and controlled release of the drug to the lung by inhalation. The in vitro drug release test and in vivo bio-distribution of CS-coated and uncoated LPs were also studied. A good deposition of CS-coated LPs was observed in the lung site that proved the efficacy for pulmonary drug delivery [72].

## 6. Chitosan Based Nanomaterials as Transdermal Nanocarriers

The transdermal route is widely studied because it has many advantages, including boosted patient compliance, tissue targeting, increased drug release, eluding the first pass metabolism, protecting drugs from gastrointestinal enzyme or acidic environment of the stomach and so forth. A great challenge of this route of administration is the penetration of the drug through the stratum corneum, which acts as a skin barrier [152]. Transdermal drug delivery systems (TDDSs) are highly attractive to researchers because it can release drugs in a controlled manner through the skin and directly into the blood stream. One of the advantages of the TDDSs is the avoidance of the first pass metabolism through the hepatic portal vein system and GIT, which represents a considerable impediment for oral administration [152]. Another benefit of TDDSs is represented by the ability to avoid loaded drug degradation by pH-associated deactivation and by enzymes, thus resulting in an efficient therapy [153]. TDDSs are non-invasive in comparison to parenteral administration, remaining relatively painless, therefore enhancing patient compliance and acceptability [152]. Nonetheless, there are various shortcomings of drug permeation due to the impermeability of the skin, especially due to the components from the structure of the stratum corneum, and as result only a small number of hydrophobic drugs, with molecular weight ≤500 Da, are able to penetrate via the transdermal administration [154,155]. For this reason, in order to enhance the ability of molecules to pass through the skin, many technologies have been developed and in the following we will give some examples of chitosan-based nanoformulations used as transdermal drug delivery systems.

### 6.1. CS-NP as Transdermal Nanocarriers

Among TDDSs, NPs take an important place based on their advantages such as increased drug encapsulation capacity of hydrophilic and hydrophobic drugs, high drug release, proper penetration across the skin barrier due to the small particle size, steady and prolonged drug release at a pre-determined rate. The three pathways for the penetration of NPs through the skin are the inter-cellular lipid route, the trans-cellular route and the follicular route. The last one seems to be the most important permeation pathway for drug delivery as a consequence of their proximity to the capillary vessels [156]. CS through a positively charged amino can interact to the negatively charged areas on the epithelial cell membranes and can unlock narrow junction improving drug diffusion to in-depth skin layers [155,156].

A recent study reported the encapsulation of lipophilic curcumin, well known for its anti-inflammatory, antioxidant, and antitumor effects, into CS-NPs for transdermal delivery. An in vitro experiment showed curcumin loaded CS-NPs having a high permeability coefficients (kp) 10.278 (cm/h) × 10^3^ and steady-state fluxes (Jss) 5.14 ± (μg/cm^2^·h), which means that CS-NPs has the ability to efficiently deliver curcumin through the corneum stratum [156]. Al-Kassas et al. [157] have prepared CS-NPs loaded with propranolol, by the ionic gelation method using tripolyphosphate (TPP) as a cross-linking agent. The ex vivo drug release study provided a high permeation through pig ear skin, which supported that CS-NPs can be a promising transdermal delivery system for propranolol [158]. Pirfenidone is the first antifibrotic agent FDA approved to treat idiopathic pulmonary fibrosis. Unfortunately, this drug has low oral bioavailability with a short half-life (2.4 h), a low absorption after oral administration and high rate of excretion through urine (80% to 85%). CS-sodium alginate NPs loaded with pirfenidone developed for transdermal route showed efficient strategy for drugs delivering through the skin into the blood flow. The in vitro release profile of pirfenidone from NPs showed a burst release of about 12% in first 5 h, but after this period of time until 25 h it was noted a sustained release. Ex vivo permeation experiments were performed using fluorescence microscopy and Franz diffusion cell, where it was reported that skin penetration of pirfenidone loaded NPs was 1/5 fold as compared to pirfenidone solution, proving that this nano-carrier can be suitable for delivery of the drug [159].

### 6.2. CS-NFs as Transdermal Nanocarriers

NFs, due to their multi-functional characteristics, offer great potential to improve/enhance localized drug delivery. The development of a novel NFs wound dressing with multi-functional properties, electro-activity, appropriate mechanical characteristics, antioxidant and antibacterial action in order to stimulate the wound healing process is desirable for the increasing requirements of clinical needs in the management of wound care [160]. 

Recent studies report the development of electroactive NFs membranes with antioxidant and antibacterial activity, using electrospinning method of quaternized chitosan-graft-polyaniline (QCSP) and poly(ε-caprolactone) (PCL) polymer solutions as wound dressing materials. The most important results were proved for QCSP15/PCL (15 wt% of QCSP in sample), which presented a balanced capacity between cell proliferation and antibacterial properties, resulting in a substantially accelerating the wound healing process in a mouse full-thickness wounds defect model in comparison with control positive sample (commercial dressing-Tegaderm™ film) and control negative sample (QCSP0/PCL nanofibrous membrane). The same formulation QCSP15/PCL nanofiber was also noted after histopathological analysis and immunofluorescence staining, where it exhibited more angiogenesis, granulation tissue thickness and higher collagen deposition [160].

Zou et al. [161] reported the successful encapsulation of the antibacterial peptide derived from king cobra, cathelicidin OH-CATH30 (OH-30) in a polyvinyl alcohol/chitosan (PVA/CS) NFs scaffold with carboxymethyl chitosan nanoparticles (CMCS-NPs), resulting nanofibrous mats with carboxymethyl chitosan cathelicidin nanoparticles (CMCS-OH-30-NPs). The effect of this formulation on the release of OH-30 and the antibacterial activities against *E. coli* and *S. aureus* were analyzed. The in vivo biological evaluation using wound model induced on specific pathogen free grade KM mice demonstrated that CMCS-OH-30-NPs induced a smoother surface coating, a more uniform epithelial surface thickness, and a more sustained connective tissue orientation in comparison with the control. These results revealed the dual beneficial wound healing and antibacterial properties of the developed NFs and can have a great potential for improving wound management with less sequelae [161].

The incorporation of natural products with well-known anti-oxidant and antibacterial effects in the NFs electrospun structure is also one of the concerns of scientists. Such a study was recently carried out by Shokrollahiet al. [162], which used *Chamomile* extract with the antioxidant, antibacterial and promoter properties of epithelial regeneration in order to develop novel biocompatible multi-layered nanofibrous patches. The composition of this nanofibrous formulation consists of 3 layers: the first outer layer of poly (ε-caprolactone) (PCL), the second layer composed of hybrid nanofibers of *Chamomile*/carboxyethyl chitosan/polyvinyl alcohol (*Chamomile*/CECS/PVA) and the *Chamomile* loaded CECS/PVA as the third interior layer. The nanofibrous multi-layered formulation is very beneficial for wound dressings because the hydrophilic *Chamomile* loaded CECS/PVA nanofibrous layer allows good contact with the wound, while the hydrophobic PCL nanofibrous layer provides the strength required for the electrospinning process. The mats showed satisfactory tensile strength (8.2–16.03 MPa) and antioxidant characteristics against DPPH radical (6.60–38.01%). The antibacterial efficiency against *E. coli* and *S. aureus* demonstrated values of inhibition zones directly proportional with the concentration of the *Chamomile* content and a better antibacterial effect than the commercial wound dressing Ag coating. The mechanism of release from the formulated NFs was Fickian diffusion-controlled, with a sustained release of *Chamomile* available for 336 h. An MTT assay revealed good cell viability for all mats except one contained 30 wt% *Chamomile.* Based on the findings from this study, the chitosan multi-layered electrospun nanofiber formulation with a 20 wt% *Chamomile* content can be appropriate for wound dressings, because of its biocompatibility, antioxidant, antibacterial and mechanical properties [162].

The recent application of CS for the production of electrospun membranes with biomedical application are depicted in Table 5. 

### 6.3. CS-NGs as Transdermal Nanocarriers

The small size, large surface area and swelling properties makes from NGs a perfect system for the efficient delivery of drugs in healing burn wounds. El-Feky et al. [175] reported CS-NGs conjugated with alginate for the delivery of silver sulfadiazine for wounds treatment. It was noted that the NGs particle size depends on silver sulfadiazine concentration, decreasing with increasing concentration, from 0.1% to 0.5%. In the CS-NGs containing 0.4% alginate and 0.5% silver sulfadiazine, the particles size was 130 ± 21 nm while for the formulation with the same concentration of alginate but 0.1% silver sulfadiazine the particle size was 980 ± 98 nm. Moreover, increasing the silver sulfadiazine concentration was associated with reduced entrapment efficiency too (entrapment efficiency: 30.80 ± 1.91%, for 0.5% silver sulfadiazine vs. 62.65 ± 2.89% for 0.1% silver sulfadiazine). Furthermore, the swelling rate generally diminished with augmentation of the silver sulfadiazine concentration (97% for 0.1% vs. 45% for 0.5%). In vivo experiments conducted on induced burn wounds on rats for a predicted NGs formula containing 0.4% alginate and 0.4% silver sulfadiazine showed that NGs proved more effective, in inferior concentrations, in a comparison made with a market pharmaceutical product. The large surface area alongside the small size of the released drug helps with infection control and in reversing wound healing impairment [175]. Abioye et al. [102] developed ternary CS-ibuprofen-gellan-NGs for controlling ibuprofen transdermal delivery, a poorly soluble drug, onto an intact skin, using an association of ionic gelation and electrostatic nano-assembly procedures. The ibuprofen embedding efficiency in the ternary NGs was slightly higher (96.67 ± 8.48%) that other polymeric delivery systems, due the electrostatic interactions between ibuprofen and CS. Rising concentrations of CS enhanced the number of hydrophobic groups accessible for interactions with ibuprofen, resulting in higher embedding efficiency and also resulting in asignificantly smaller particle size of NGs (14.15 ± 2.39 nm) and to decreased swelling ratio. The ex vivo release assays performed on pig skin indicated that the CS-ibuprofen-gellan conjugate NGs are more permeable than ibuprofen-gellan hydrogel used as a control. So, it was considered that the *ternary* CS-ibuprofen-gellan NGs enhanced the ibuprofen release thru the skin by ameliorating skin penetration, skin retention, percutaneous drug delivery due to the conversion of crystalline drug into amorphous particles and due to the particle size diminution [102].

### 6.4. CS-LPs as Transdermal Nanocarriers

The research of Mengoni et al. [176] revealed that some chitosan-coated LPs loading substance P (SP) could have potential applications for wound healing. It was reported that SP presents a vasodilator effect, angiogenesis or release of nitric oxide promoter activity. The liposome formulation proved to possess a higher efficacy and a larger therapeutic interval than free SP, probably due to the prolonged release of SP. This SP-delivery system could be used in treating difficult chronic wounds such as diabetic wounds [176].

Lee et al. [177] developed chitosomes encapsulating indocyanine green in order to stabilize and enhance skin permeation. Indocyanine green has proven to be a promising candidate for the topical melanoma-photodynamic therapy. The results evidenced the protection from degradation of the incorporated drug, increasing of skin permeation, cellular uptake and photo-cytotoxicity on B16-F10 melanoma cells of the indocyanine green.

## 7. Advanced CS Based Nanomaterials as Targeted Drug Nanocarriers

### 7.1. CS Based Nanocarriers for Gene Delivery

Gene delivery opens up a promising direction in the treatment of various fatal diseases, especially cancer. Gene delivery systems are designed to target a specific cell, to enhance the delivery of genes and to provide a sustainable-release of the gene. The formulation of the gene delivery system must also ensure protection of the gene from nuclease degradation and escape capability from the lysosome, and must have low cytotoxicity and high transfection efficiency [178]. The delivery of small DNA (DNAs) and short interfering RNA (siRNA) are imperious tools in cancer therapy, to silence target gene expression [179]. The ability of CS to be used for DNAs/siRNA delivery is mainly based on the combinations of hydrogen and ionic bonds. The positively charged surface of CS can complex with the negatively charges of gene to form stable polyplexes. The stability of polyplexes is correlated with the ability to protect DNA from nuclease degradation [53].

siRNA was formulated as CS-NPs to protect siRNA from the gene segmentation enzymes, from gastric breakdown and transport it to the tumor cells through an intracellular compartment [143]. In the experiment conducted by Ballarín-González et al. [180] it was observed that, after oral gavage in mice with siRNA NPs, it was deposited in much greater quantities along the stomach, small and large intestine compared to siRNA administration, where rapid nucleic acid degradation was recorded [180,181]. The DNA and siRNA can be incorporated also into CS-NGs once these are already formed, resulting in adsorption of the negatively charged molecule on the positively charged surface of the NGs [182].

Recently, by chemical attachment of folate-conjugated poly(ethyleneglycol) (PEG) to glycol-CS-NGs, the nanomaterials obtained with an average size environ of 200 nm, were able to avoid the phagocytosis by macrophages due to the PEG, also having increased time to circulate in the bloodstream [178]. The ligand provided a good internalization of the NGs in the tumor targeted cell such as HeLa cells, through folate receptor-mediated endocytosis. A tight and stable interaction between glycol-CS-NGs and siRNA was observed at an amine phosphate ratio of 10 [183].

Another tumor target for gene delivery is the protein that is indispensable for tumor growth and metastasis, the vascular endothelial growth factor (VEGF) [184]. Liu et al. [98], developed a pH-responsive CS-agmatine conjugates incorporating VEGF- suppressing siRNA. The NGs designed initial as negative surface charge, were stable under physiological condition (pH 7.4) and provided prolonged blood circulation kinetics. The decrease in pH value at the tumor site subsequently provided a reverse in the NGs surface charge. The zeta potential of the NGs was promptly inverted within 30 min from −12 to +9 mV when the pH was lowered from 7.4 to 6.5. The charge conversion of the VEGF-suppressing siRNA loaded CS-agmatine conjugates NGs in the tumor extracellular medium offered an enhanced target affinity for the negatively charged cell membrane which contributes to cellular intake and endo/lysosomal escape. Investigating the effects on Hela tumor-bearing nude mice after intravenous administration showed that siRNA loaded charged-conversional NGs successfully suppressed VEGF expression and micro-vessel growth together with effectively inhibiting tumor cell proliferation in comparison with the CS/VEGF-siRNA complexes [184,185].

Based on the cumulative efficiency of siRNA, pH-sensitive CS liposomes for co-delivery of sorafenib (Sf) and siRNA were developed as new antitumor therapeutic systems. Tumor accumulation and antitumor activity were evaluated by the Kunming mice H22 cells-bearing tumor model. The results evidenced that this system developed a high Sf release and siRNA tumor accumulation compared to blank siRNA. A lower toxicity and an increased Sf accumulation were also noted in tumor cells [186].

### 7.2. CS Based Nanocarriers for Antitumor Drug Delivery

In the cancer therapy, the release of anticancer drugs precisely and specifically at a target sites is still a challenge. The design of stimuli-responsive NGs (such as enzymes, temperature, pH and redox potential) provides hope for controlling drug release at tumor tissues. The pH values differentiation between extracellular medium of the tumors (pH 6.5–7.2), the normal tissues and blood (pH 7.4) and the pH values of lysosomes and endosomes (pH 5.0–6.5) supports the research on pH-responsive NGs development. Several cancer drugs such as doxorubicin [65,187], 5-fluorouracil [66], paclitaxel [188], bleomicyn [67], oridonin [189] were embedded into CS-NGs.

A pH-responsive glycol CS-NGs grafted with functional 3-diethyl-aminopropyl (DEAP) groups loaded with doxorubicin (DOX) were developed. The NGs was stable at physiological pH and at slightly acidic pH condition in presence of non-small lung carcinoma A546 cells was imbalanced as a result of the DEAP protonation. DOX release was accelerated, which increased its uptake in the tumor cells [65]. Indulekla et al. [190] designed magnetic NGs for an efficient release control of DOX. In order to obtain a dual, thermo and pH, responsive NGs, the CS (low molecular weight) pH responsive properties were combined with poly (*N*-vinylcaprolactam), known as thermo-response polymer. The magnetic nanoparticles based on iron oxide, encapsulated into the hybrid NGs provide increase of local heat in presence of magnetic field. The in vitro drug release study demonstrated that the magnetic field produced hyperthermia correlated with significantly higher drug release (∼73%). In addition, the amount of DOX could be controlled by mode ON/OFF trigger of the alternating current magnetic field. DOX release was also considerably higher (∼58%) at pH 4.5 and above 43 °C within 1 in comparison with all other release conditions (pH7.4, 37 °C) [190].

Another CS-NGs stabilized with plutoniu 127 was designed for a controlled and sustainable release of bleomycin, targeting the skin cancer. This CS-NGs showed a high entrapment efficiency for bleomycin (55%) with an insignificant release profile at normal skin cell pH but with an enhance release at moderately acidic medium (pH 5–6) and with a sustained release profile during 24 h [191]. The substitution of CS with galactose improved the antitumor activity of oridonin targeting the liver cancer cells. The pH responsive galactosylated-CS-graft-poly (*N*-isopropylacrylamide) loaded with oridonin exhibited a higher antitumor activity than drug-loaded NGs without galactosylation, and the anticancer action increases in direct proportion with the number of galactose moieties of the NGs in HepG2 cells. These NGs enhance the uptake of oridonin into HepG2 cells via asialoglycoprotein receptor-mediated endocytosis [189].

Liang et al. [192] have reported the pharmacokinetic behaviour and antitumor effect of new N-palmitoyl chitosan anchored docetaxel liposomes (NDLPs) compared with plain docetaxel liposomes (PDLPs) and PEGylated docetaxel liposomes (PEGDLPs). Docetaxel is one of the most important chemotherapeutic agents against ovarian carcinoma and breast, lung and head/neck cancer but unfortunately presents some side effects. The inclusion of docetaxel in liposomes targeted the decreasing of its toxicity and clearance. The studies which were focused on determination of docetaxel concentration in rat plasma after intravenous administration using RP-HPLC, revealed that NDLPs demonstrated higher concentrations than PDLPs, but not higher than PEGDLPs. Docetaxel liposomes exhibited an increased stability, a significant increase of the half-life and a decrease of clearance that revealed a slow drug release by protection of the lipid bilayer membranes [192].

Paclitaxel loaded chitosan and acylated(myristoyl and octanoyl) chitosan coated liposomal formulations were reported by Nanda et al. [193]. It was noted that liposomes coated with acylated chitosan presented slower drug release in comparison to uncoated and chitosan coated liposomes. All the liposomal formulations also proved to be less cytotoxic than paclitaxel injection. The myristoyl chitosan coated liposomes revealed increased pharmacokinetic, biodistribution and tumor uptake features towards the rest of the formulations. These findings may confirm the real potential of these liposomal delivery systems in the tumor targeting of paclitaxel [193].

## 8. Conclusions and Future Trends

Nanotechnology has attracted considerable interest from scientists in biomedical and pharmaceutical applications and, with the advances in biopolymer science, a large number of multifunctional nano-materials based on CS were formulated as DDSs. CS as a biocompatible, biodegradable, non-toxic polymers and with interesting biological properties plays a key role in the development process of new controlled and targeted DDSs. This article targeted the essential characteristics of CS-based nano-materials used as nanocarriers in the drug delivery medicinal area, such as oral, transmucosal, pulmonary, transdermal or targeted drug delivery. For a better understanding of the release of various active substances incorporated into the reviewed CS based formulations (NPs, NFs, NGs, LPs), a description of the particularities and advantages of the specific route of administration was made. The advantages or the draw-backs of these CS multifunctional nano-materials acting as drug delivery systems or targeted drug delivery systems were also highlighted. Despite the potential progression achieved in the nanotechnology domain, CS based nano-formulations have not yet made an efficient transition into the market, highlighting the necessity for further research to improve the existing shortcomings. There is the need to test the new developed chitosan nano-mats in suitable animal models and to make the comparison with the products that already exist in the pharmaceutical market in order to demonstrate the suitability for clinical trials. Another idea to be deepened in the future is that of scaled-up nanotechnologies for the cost-effective and industrial-scale fabrication of nano-formulations suitable for the delivery of drugs through different mucosa.

## Figures and Tables

**Table 1:** The most important characteristics of different CS-NPs (selection).

CS-NPs	NPs Size (nm)	DLE (%)	DR (%)	Mw/DD of CS	ZP (mV)	Ref.
Insulin CS-NPs	534 ± 24	80 ± 3.96%	14%(pH 2) 85–88% (pH 6.8) (after 10 h)	CS LMw 150 kDa DD 95%	14.57 ± 1.1	[27]
Docetaxel solid-lipid CS-NPs	235 ± 4.2	94 ± 3.1%	84 ± 3.1% (donor: acceptor lipid-1:25) 88 ± 2.5% (donor: acceptor lipid-1:100)	CS HMw 310 kDa DD 75%	29.0 ± 3.5	[31]
Sodium ceftriaxone CS-NPs	265 ± 3.5	79 ± 0.9%	52% (after 24 h) 58% (after 48 h)	CS MMw 190–310 kDa DD 87%	45.27 ± 2.1	[29]
Dexketoprofen- Trometamol CS-NPs	726.8 ± 16.8	732 ±1.2%	93.10 ± 7.07% (after 48 h)	CS LMw 50–190 kDa DD *	53.3 ± 2.2	[25]
Erlotinib CS-NPs	170.2 ± 2.9	74.45 ± 0.3%	89.46% (after 24 h)	CS LMw 40–80 kDa DD 95%	16.2 ± 1.2	[32]
Simvastatin CS-NPs	113 ± 4.9	97.70 ± 0.1%	98.60% ± 0.40% (after 14 days)	CS LMw 50–190 kDa DD ≥ 85%	40.80 ± 0.1	[30]
Sumatriptan succinate CS-NPs	105 ± 10.1	59.60 ± 2.1%	68.03 ± 3.98% (after 72 h)	CS LMw 40–80 kDa DD *	21.5 ± 1.0	[24]

* unspecified.

**Table 2:** The most important characteristics of different CS-NFs (selection).

CS-NFs	DC	DR (%)	Mw/DD of CS	Ref.
Donepezil CS/PVA-NFs	5 mg in 40 mg CS and 125 mg PVA	97% (after 10 min)	CS LMw 50–190 kDa DD *	[44]
Ranitidine hydrochloride CS/PEO-NFs	0.15 mg/mL polymeric solution	40% (pH-responsive, burst release after 2 h)	CS MMw 1000 kDa DD *	[45]
Naproxen CS-NFs	30% of the membrane mass	90% (burst release after 10 min)	CS LMw 60–120 kDa DD *	[46]
Sumatriptan succinate CS-NFs	20% of the membrane mass	90% (burst release after 10 min)	CS LMw 60–120 kDa DD *	[47]
Tetracycline hydrochloride CS/PVA-NFs	3 μg/mL at 2 mg NFs	80% (burst release after 2 h)	CS MMw Mw * DD 75–85%	[42]
Cisplatin CS-NFs	98.6 ± 1%	30% (burst release, after 10 days) 69.6% (steady state release, after 30 day)	CS HMw 310 kDa DD *	[47]
N-IpaD antigen CS-NFs	64.7 ± 14.3%	99% (after 2.5 h)	CS HMw 375 kDa DD 75–85%	[48]

* unspecified.

**Table 3:** The most important characteristics of different CS-NGs (selection).

CS-NGs	DC/DLE (%)	DR (%)	Mw/DD of CS	Ref.
Myricetin CS-NGs	1.33 mg/mL of polymeric mass	83% (after 4 h, pH 1.2) reached the equilibrium state after 12 h	CS LMw 20 kDa DD 90%	[63]
Triclosan/ Flurbiprofen CS-NGs	DLE: Triclosan (93.67 ± 3.51%), Flurbiprofen (96.33 ± 2.08%)	80% (burst release in first 2 h)	CS MMw 190–310 kDa DD 84%	[60]
Doxorubicin CS/CMCS-NGs	0.5 mg/mL with DLE of 71.84 ± 3.1 %	200 ng/mL in vivo release in plasma (after 7 h)	CS LMw, 10 kDa DD 89%; CMCS MMw, 12 kDa, DD 81%, DS 92%	[64]
Doxorubicin GlyCS-NGs	2 mg/mL with DLE of 78 ± 3.1	23% (pH 6.8) and 8% (pH 7.4) after 4 h; 20% (pH 7.4) and 59% (pH 6.8) after 24 h	Gly CS Mw 250 kDa, DD 82.7%	[65]
5-Fluororuacil CS/PLGA-NGs	DLE of 39 ± 0.2% in CS-NGs	25–30% (pH 7.0), after 24 h 70–85% (pH 6.0), after 24 h	CS MMw Mw * DD 75%	[66]
Bleomycin CS-NGs	DLE of 54.0 ± 0.95% in CS-NGs	35% (pH 7.0), 55% (pH 4.0), 85% (pH 6.0), after 24 h	CS MMw Mw * DD 75%	[67]

* unspecified.

**Table 4:** The most important characteristics of different CS-LPs (selection).

CS-LPs	LPs Size (nm)	DLE (%)	DR (%)	Mw/DD of CS	ZP (mV)	Ref.
Curcumin hybrid MCS-LPs	54.1 ± 2.4	8.08 ± 0.18%	13.1% (after 12 h) 15.5% (after 24 h)	CMCS Mw 10 kDa DD 85%	26.3 ± 2.3	[73]
Glutathion/Ferulic acid CS-LPs	460.3 ± 6.0	Gluthation: 61.32 ± 1.32; Ferrulic acid: 68.92 ± 1.27	-	CS LMw 100 kDa DD ≥ 95%	57.7 ± 1.3	[75]
N acetyl Cys CS-LPs (DPPG 5% CS: lipid ratio 1:1)	610.08 ± 8.3	74 ± 1.73%	38% (after 7 h)	CS Mw 110–150 kDa DD ≤ 40%	38.1 ± 0.9	[72]
Acteoside CS-LPs	92.77 ± 2.99	88.10 ± 5.36%	54.82% (after 4 h) 67.34% (after 8 h)	CS LMw 100 kDa DD ≥ 95%	19.65 ± 0.9	[74]
Triamcinolone acetonide CS-LPs	100.3 ± 6.8	98 ± 5.36%	-	CS LMw 100 kDa DD *	31.2 ± 0.8	[81]
Curcumin thiolated CS-LPs	406.0 ± 12.0	93.95 ± 3.94%	40.39% (pH 5.5, after 12 h); 24.93% (pH 7.4, after 12 h)	CS MMw, DD 78%	36.6 ± 0.6	[76]

* unspecified.

**Table 5:** Recent data on CS-based electrospun NFs used as transdermal nanocarriers.

Formulation	Active Substance/ Extract Embedded	Applications	Ref.
CS/PEO electrospun wound scaffold	*Aloe vera* extract	wound dressing	[163]
biomimetic nanocomposite scaffolds based on surface modified PCL-CS/gelatin NFs	Curcumin	skin regeneration	[164]
CS/PEO NFs	Bromelain (crude extract from pineapple)	burn wound healing in animal model	[165]
electrospun CS/PVA/bioglass nanofibrous membrane	**-**	wound dressings for promoting healing of chronic wounds	[166]
electrospun PLA CS core-shell NFs	Curcumin	wound dressing and drug delivery	[167]
composite aliphatic copolyamide /PEO/CS based on Chitin/CS-NFs	Chitin nanofibrils	wound dressing for treatment of third-degree burns	[168]
HA coated electrospun CS/PEO-based NFs	**-**	tissue engineering	[40]
bilayer CS NF scaffold based on mammalian gelatin and fish collagen	*Lithospermi radix* extract	wound healing in a rat model	[169]
PEO-CS-NFs	Ciprofloxacin, zinc oxide	burn wounds management	[170]
electrospun PVA-CS based NF mats	*Zataria* multiflora essential oil	antimicrobial wound dressings	[171]
CS/alginate nanofibrous wound dressing	Gentamicin	drug delivery systems and skin regeneration	[172]
CS/PVANFs	Silk protein sericin	wound dressing	[173]
reinforced CS-NFs	nanocrystals of cellulose -graft-poly (*N*-vinyl caprolactam)	skin tissue engineering	[174]
